# Inclusion of cancer-associated fibroblasts in drug screening assays to evaluate pancreatic cancer resistance to therapeutic drugs

**DOI:** 10.1007/s13105-021-00857-2

**Published:** 2021-12-05

**Authors:** Sarah Brumskill, Lawrence N. Barrera, Peter Calcraft, Caroline Phillips, Eithne Costello

**Affiliations:** 1 Institute of Translational Medicine, Department of Molecular and Clinical Cancer Medicine, University of Liverpool, 2nd Floor Sherrington Building, Ashton Street, Liverpool, L69 3GE UK; 2 Redx Oncology, Alderley Park, Macclesfield, Cheshire UK

**Keywords:** CAFs, PDAC resistance, Drug screening, PDAC chemotherapy

## Abstract

Pancreatic ductal adenocarcinoma (PDAC) is characterised by a pro-inflammatory stroma and multi-faceted microenvironment that promotes and maintains tumorigenesis. However, the models used to test new and emerging therapies for PDAC have not increased in complexity to keep pace with our understanding of the human disease. Promising therapies that pass pre-clinical testing often fail in pancreatic cancer clinical trials. The objective of this study was to investigate whether changes in the drug-dosing regimen or the addition of cancer-associated fibroblasts (CAFs) to current existing models can impact the efficacy of chemotherapy drugs used in the clinic. Here, we reveal that gemcitabine and paclitaxel markedly reduce the viability of pancreatic cell lines, but not CAFs, when cultured in 2D. Following the use of an in vitro drug pulsing experiment, PDAC cell lines showed sensitivity to gemcitabine and paclitaxel. However, CAFs were less sensitive to pulsing with gemcitabine compared to their response to paclitaxel. We also identify that a 3D co-culture model of MIA PaCa-2 or PANC-1 with CAFs showed an increased chemoresistance to gemcitabine when compared to standard 2D mono-cultures a difference to paclitaxel which showed no measurable difference between the 2D and 3D models, suggesting a complex interaction between the drug in study and the cell type used. Changes to standard 2D mono-culture-based assays and implementation of 3D co-culture assays lend complexity to established models and could provide tools for identifying therapies that will match clinically the success observed with in vitro models, thereby aiding in the discovery of novel therapies.

**Supplementary Information:**

The online version contains supplementary material available at 10.1007/s13105-021-00857-2.

## Introduction

Pancreatic cancer is the 4^th^ leading cause of cancer deaths [52] and has an overall 5-year survival of 7–9% [13, 48]. Poor survival is due in part to a lack of effective therapies with many clinical trials of potential PDAC treatments failing [6]. A feature of PDAC, which presents a significant barrier to therapeutic intervention, is its dense tumour microenvironment [56].

PDAC-associated fibroinflammatory stroma harbours a complex array of activated cancer-associated fibroblasts (CAFs), immune cells, endothelial cells and extracellular matrix proteins such as collagen and fibronectin [42, 44, 59]. The most abundant cell type in PDAC stroma is CAFs, which were first described in 1998 [2, 4]. These cells drive the fibroinflammatory reaction through the deposition of extracellular matrix [20] and the secretion of growth factors and cytokines [19, 51]. CAFs also play a significant role in chemoresistance through a variety of mechanisms. For example, the extracellular matrix deposited by CAFs forms a physical barrier to chemotherapy and mediates increased interstitial fluid pressure within the tumour causing impaired vascular function [25]. Additionally, CAFs have been reported to scavenge gemcitabine, a nucleoside analogue, used to treat PDAC, thus reducing the availability of the drug to cancer cells [23]. They therefore should be considered early in the drug development process in order to maximise the probability of clinical success.

Gemcitabine was approved for use in metastatic PDAC in 1996 [10, 41] and is still used today for the treatment of patients with resectable or advanced PDAC, either as a single agent or in combination with other agents [30, 50]. One such other agent is paclitaxel, a microtubule stabilising molecule which has been used to treat many solid tumours. The combination of gemcitabine and nanoparticle albumin–bound paclitaxel (nab-paclitaxel) is a standard of care option for patients with advanced disease [38, 57].

The failure rate of drugs tested in phase III clinical trials for PDAC is high, with a large discrepancy between the behaviour of drugs or drug combinations pre-clinically and their performance in randomised control clinical trials [55]. Traditional 2D monolayer-based assays have played a key role in the drug discovery process for decades and are still in use as they provide a cost-effective tool that is fast and easily adapted for high-throughput screening strategies [24, 36]. Novel organotypic cell culture models and 3D spheroid cultures containing pancreas-derived CAFs have been developed in order to investigate the complex interactions between CAFs and PDAC tumour cells [17, 43]. Such models, however, have not been incorporated routinely into drug testing programmes. Here, we sought to determine whether the inclusion of CAFs to cell-based assays or whether altering the dosing schedule or the format of the cell culture would affect the ability of gemcitabine paclitaxel to kill PDAC cells in vitro and better reflect clinical efficacy.

## Materials and methods

### Specimen collection

Patients with PDAC were selected for this project. Fibrous appearing tissue was selected by the pathologist after a gross analysis of the specimen.

### Isolation of CAFs

CAFs were isolated previously [7] using the outgrowth method [2, 4]. This method involves the explantation of pieces of fibrotic tissue removed from the pancreas. Briefly, small pieces of tissue 1 mm are arranged in uncoated 6-well tissue culture plates (5–7 pieces/well), and 500μL of Iscove’s modified Dulbecco’s medium (IMDM) supplemented with 20% fetal bovine serum (FBS), 2% L-glutamine and 1% penicillin–streptomycin was added carefully to ensure that the tissue pieces were not disturbed. Plates were incubated overnight at 37 °C in a 5% CO_2_ air humidified atmosphere. The tissue pieces were removed when fibroblasts reached about 30% confluence. The medium was changed twice weekly using the same medium formulation described above, and cells were grown to 80% confluence, harvested and stored in liquid nitrogen.

### Cell culture

Cell lines were purchased from the American Type Culture Collection ATCC (MIAPaCa-2, PANC-1, Suit-2, BxPC-3 and AsPC-1). Pancreatic cancer cell lines were cultured in Dulbecco’s modified Eagle’s medium (DMEM GlutaMAX) supplemented with 10% FBS. Primary CAFs were recovered from liquid nitrogen simultaneously and placed in a T75 flask with IMDM supplemented with 10% FBS, 2% L-glutamine and 1% penicillin–streptomycin. Both primary and established cells were incubated at 37 °C in a 5% CO_2_ air humidified atmosphere. Cells were validated by STR profiling and tested for mycoplasma using e-Myco plus mycoplasma PCR detection kit (iNTRON Biotechnology) following the manufacturer’s instructions.

### Immunofluorescence

Immunofluorescence (IF) staining was used to stain 2D co-cultures of CAFs and epithelial cancer cell lines. 2D co-cultures CAFs and PANC-1 cells were seeded using IMDM supplemented with 10% FBS, 1% L-glutamine and 1% penicillin–streptomycin in 96-well, black, clear bottom, cell carrier plates (PerkinElmer, UK) and left for 24 h to adhere; mono-cultures were also seeded for comparison. The cells were dosed with gemcitabine using a D300 digital liquid dispenser (Tecan). After 72 h, the media was removed from the plates, and the cells were washed twice with PBS ensuring the complete removal of liquid from the wells each time. The cells were fixed with 100μL of 4% PFA per well, for 10 min at room temperature before progressing onto the staining protocol. The samples were permeabilised with 0.1% (v/v) Triton X-100 in PBS and incubated for 30 min at room temperature. The plates were then washed twice for 5 min in PBS and blocked with 5% (v/v) goat serum in PBS for 1 h at room temperature before the addition of primary antibodies diluted in blocking solution.

Cells were incubated with primary antibodies (Mouse monoclonal (1A4) to αSMA, Rabbit polyclonal to wide spectrum cytokeratin) or isotype controls overnight at 4 °C. The following day, the antibodies were removed, and the plates were washed three times with PBS for 5 min each. Secondary antibodies (Goat Anti-Mouse IgG H&L (Alexa Fluor® 488, Goat Anti-Rabbit IgG H&L (Alexa Fluor® 594))) were diluted at 1:500 and incubated for 1 h at room temperature. The secondary antibodies were removed, and the nuclei were stained with 4’, 6-diamidino-2-phenylindole (DAPI) at a 1:10,000 dilution for 10 min at room temperature. The plates were then washed 3 times for 5 min in PBS. The staining was visualised using an Operetta High-Content Imaging system (PerkinElmer).

Using dual staining within the direct co-cultures, it was possible to differentiate cancer cells (CTK stained) from CAFs (αSMA stained). Using the isotype controls to remove background fluorescence, it was possible to distinguish αSMA-positive cells from CTK-positive cells. αSMA staining was used to determine CAFs which were then subtracted from the total nuclei count. Five regions were imaged in each well at 10 × magnification, with each condition run in duplicate. The data was presented as a percentage of the DMSO control.

### Collagen 1a1 ELISA

CAFs and PDAC cells were seeded in triplicate at a density of 10,000 cells/well in a 12-well plate and after 72 h supernatant was harvested and centrifuged at 400 g for 5 min to remove cell debris. In order to quantify the collagen1a1 present in the supernatant a Human Pro-Collagen 1 alpha 1 DuoSet ELISA (R&D Systems) was used following the manufacturer’s instructions. Absorbance was measured using an EnVision multilabel plate reader (PerkinElmer) at 450 nm.

### 2D proliferation assay

Cells were seeded on 96-well flat, white, clear bottom plates (Greiner Bio-One) at 1000 cells/well in 100µL of DMEM-GlutaMAX supplemented with 10% FBS and left for 24 h to adhere. The following day, cells were treated with either gemcitabine or paclitaxel using a D300 digital liquid dispenser (Tecan). The plates were placed in an incubator (37 °C, 5% CO_2_) for 72 h. CellTitre-Glo was prepared using the manufacturer’s instructions. At the appropriate time point, plates were removed from the incubator and allowed to equilibrate to room temperature for 30 min. Ten microlitres of CellTitre-Glo reagent was added to each well, and the plates were sealed with a black plate seal and placed on an orbital shaker for 10 min. Luminescence was read using an EnVision plate reader (PerkinElmer). The luminescent signal generated is in direct proportion to the amount of ATP in the well, which is required for the conversion of luciferin to oxyluciferin in the presence of assay reagents. Cell viability was determined and compared to a DMSO control (0.1%) which was set at 100%.

### Chemotherapy pulsing

Chemotherapy pulsing was used to determine the effect of mimicking clinical dosing of chemotherapeutic agents in an in vitro assay on the viability of pancreatic cancer cell lines. The cMAX (maximum serum concentration of drug in humans) of gemcitabine (74.4 ± 11.3 μM) and paclitaxel (4.5 ± 0.4 μM) [16] was used to calculate an approximate exposure time, such that it matched the total exposure determined by the area under the curve AUC. Once cells had been incubated overnight in order to adhere, the drug was pulsed onto the cells for the length of time each drug is present in serum at a given concentration (Table 1). Once the pulse was completed, media was replaced with fresh vehicle (DMSO) containing media.

**Table 1:** Experimental conditions of chemotherapy pulsing experiment

Chemotherapeutic	0.5 cMAX		0.1 cMAX	
	Concentration (µM)	Time (min)	Concentration (µM)	Time (min)
Gemcitabine	37.21	50	7.44	250
Paclitaxel	2.25	420	0.45	2100

### Transwell co-culture model

CAFs and PANC-1 cells (1:1 ratio) were seeded onto a 96-well 0.4 µm transwell plate with 500 CAFs in the lower chamber and 500 PANC-1 in the upper chamber in IMDM supplemented with 10% FBS, 2% L-glutamine, and 1% penicillin–streptomycin. To control for cell number, transwell plates containing PANC-1 cells alone were seeded in both chambers. Cells were incubated for 24 h to adhere. The following day, they were treated with gemcitabine using a digital liquid dispenser D300 (Tecan). After 72 h, cell viability of PANC-1 cells was measured using the CellTiter-Glo.

### 3D cell culture model

Cells were seeded, either as a mono-culture or co-culture using a 1:1 ratio (500 CAFs: 500 cancer) in Ultra-Low Attachment (ULA) plates at 1000 cells/well and incubated for 24 h to form spheroids. The cell number and drug incubation were determined in PANC-1 cells using a cell density gradient assay over a number of time-points (48, 72 and 96 h) to determine the most optimal assay conditions. The cells were treated with gemcitabine or paclitaxel and incubated for 72 h. The cell viability of 3D spheroids of pancreatic cancer cell lines and/or co-cultures was determined using the Promega 3D CellTiter-Glo assay. Briefly, the CellTiter-Glo 3D reagent was thawed at 4 °C overnight and then placed in a 22 °C water bath for 30 min before use. The plate containing the spheroids was equilibrated for 30 min at room temperature. Fifty microlitres of CellTiter-Glo 3D was added to each well and placed on an orbital plate shaker for 5 min. The cell lysate and CellTiter-Glo reagent were moved to a flat, clear bottom 96-well plate, incubated for a further 25 min (room temperature), and then the luminescent signal was measured using an EnVision multilabel plate reader (Perkin Elmer).

### Statistical analysis

Statistical tests were performed using GraphPad software V.6.01. Results were considered significant at *P* ≤ 0.05.

## Results

### Gemcitabine and paclitaxel markedly reduce the viability of pancreatic cell lines, but not CAFs, when cultured in 2D

**Fig. 1:**
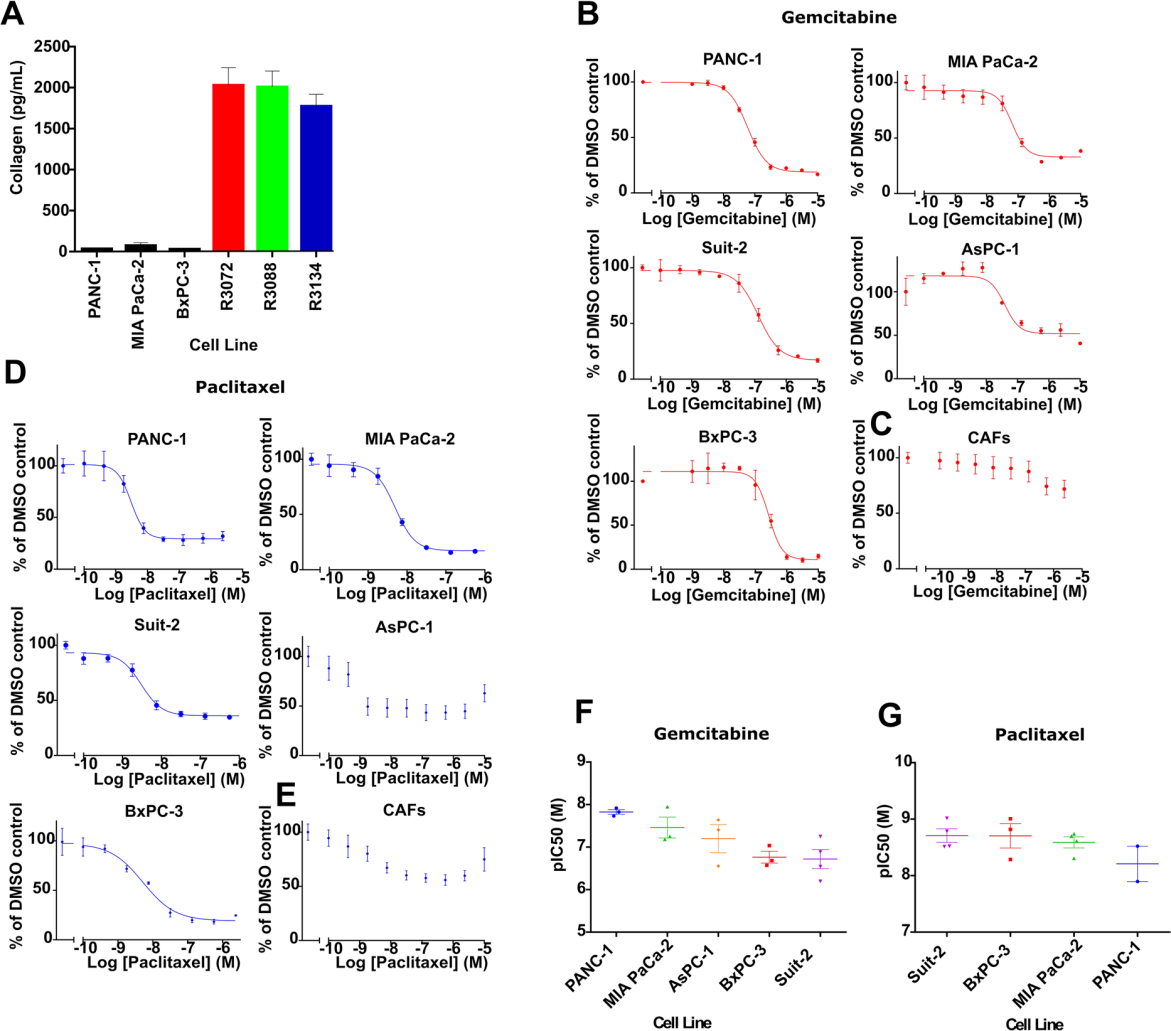
Isolated CAFs secrete collagen and are insensitive to gemcitabine and paclitaxel treatments compared to pancreatic cancer cell lines in standard 2D culture conditions. **(A)** Col1a1 secretion from three established pancreatic cancer cell lines and CAFs isolated from three different patients (R3072, R3088 and R3134) was determined by ELISA. The level of Col1a1 secreted by the cancer cell lines was below the acceptable limit of detection for the assay, precluding statistical analysis. **(B–E)** Dose–response curves following gemcitabine treatment of pancreatic cancer cell lines **(B)** and CAFs (R3072) **(C)** or paclitaxel treatment of pancreatic cancer cell lines **(D)** and CAFs (R3088) **(E).** Cells were exposed to increasing concentrations of gemcitabine or paclitaxel and cell viability measured at 72 h using CellTiter-Glo. Data were fitted to a sigmoidal dose–response curve, and IC50 was determined using GraphPad prism. For PDAC cell lines, the data are representative of three independent experiments ± SD performed in triplicate and normalised to a DMSO control set to 100%. For CAFs, the data are representative of three independent experiments using three biological replicates (R3088, R3072 and R3134) ± SD performed each in triplicate and normalised to a DMSO control set to 100%. **(F–G)** Graphs depicting the mean pIC_50_ in molar (M) ± SEM for multiple assays of gemcitabine and paclitaxel in pancreatic cancer cell lines

CAFs were isolated from PDAC patients using the outgrowth method [2, 4] and were shown previously to express the CAF markers, αSMA, desmin and vimentin, and to harbour wild-type *KRAS* [7]. In addition, to establish their functionality, we investigated their ability to secrete collagen, a major component of the PDAC microenvironment, which CAFs are responsible for depositing [20]. Conditioned media from three independent CAF isolates (R3088, R3072 and R3134) were evaluated for collagen 1a1 levels by ELISA (Fig. 1A). All three isolates secreted considerably more collagen than three established PDAC cell lines tested for comparison (Fig. 1A).

We next sought to establish the effects of gemcitabine and paclitaxel on the viability of PDAC cell lines and CAFs in a traditional 2D screening model. Five PDAC cell lines, PANC-1, MIA PaCa-2, Suit-2, AsPC-1 and BxPC-3, showed sub-micromolar sensitivity, in a concentration-dependent manner, to 72 h treatment with gemcitabine (Fig. 1B). By contrast, CAFs (R3072) were considerably less sensitive to gemcitabine with over 50% of cells remaining viable following exposure to the highest concentration (10 µM) of gemcitabine (Fig. 1C). Four of the five PDAC cell lines tested (PANC-1, MIA PaCa-2, BxPC3-3 and Suit-2) were more sensitive to paclitaxel treatment (Fig. 1D) than CAFs (R3088; Fig. 1E). More than 50% (R3088) remained viable following 72 h exposure to the highest concentration (10 µM) of paclitaxel (Fig. 1E). The viability of PDAC cells following 96 h exposure to both drugs was determined, with sensitivities to both gemcitabine (Supplementary Fig. 1A) and paclitaxel (Supplementary Fig. 1B) recorded. Across all of the experimental conditions, the observed potencies to both drugs were in the nanomolar range (Fig. 1F, G and Supplementary Fig. 1C,D), with the exception of AsPC-1 cells which showed low sensitivity to paclitaxel after 72 h (Fig. 1D). Taken together, our data indicate that isolated primary PDAC-associated CAFs retain physiological function, as attested by secretion of collagen, and are resistant to gemcitabine and paclitaxel in a standard 2D mono-culture-based assay.

### Chemotherapeutic pulsing as an alternative method for dosing cells in vitro

Drug screening assays in the pharmaceutical industry are designed with a variety of factors in consideration such as pharmacological relevance, reproducibility, quality and importantly costs [27]. In PDAC research, the standard 2D mono cell-based assay is still the most commonly used method to investigate the effect of chemotherapeutic agents on cancer cells in culture [3, 32]. In a 2D mono-culture-based assay, chemotherapeutic agents are ranked based on their potency following continuous exposure of cells to a compound. In vivo however, the length of time cancer cells are exposed to the drug varies based on a variety of factors, one of which is the rate at which the drug is cleared by the host. In order to investigate whether an in vitro dosing schedule which mimics the clinical exposure of cancer cells to the drug would better reflect the in vivo performance of the drug, a pulse experiment was designed. This involved adding the drug for a defined time (pulse) and then removing it for the remaining duration of the experiment. The cMAX values (maximum serum concentration of drug in humans) of gemcitabine (74.4 ± 11.3 μM) and paclitaxel (4.5 ± 0.4 μM) respectively [16] were used to calculate the durations of the pulses such that they matched the total exposure time and concentration determined by the area under the plasma drug concentration curve (AUC) for these agents [16]. The abundant stroma, hypo-vascularity, vascular collapse and high interstitial fluid pressure associated with pancreatic tumours impair drug delivery [45–47]. Dosing using the cMAX would therefore likely overestimate the drug exposure of a pancreatic tumour, and consequently 0.5 cMAX and 0.1 cMAX were chosen as a high and low dose respectively of gemcitabine or paclitaxel.

**Fig. 2:**
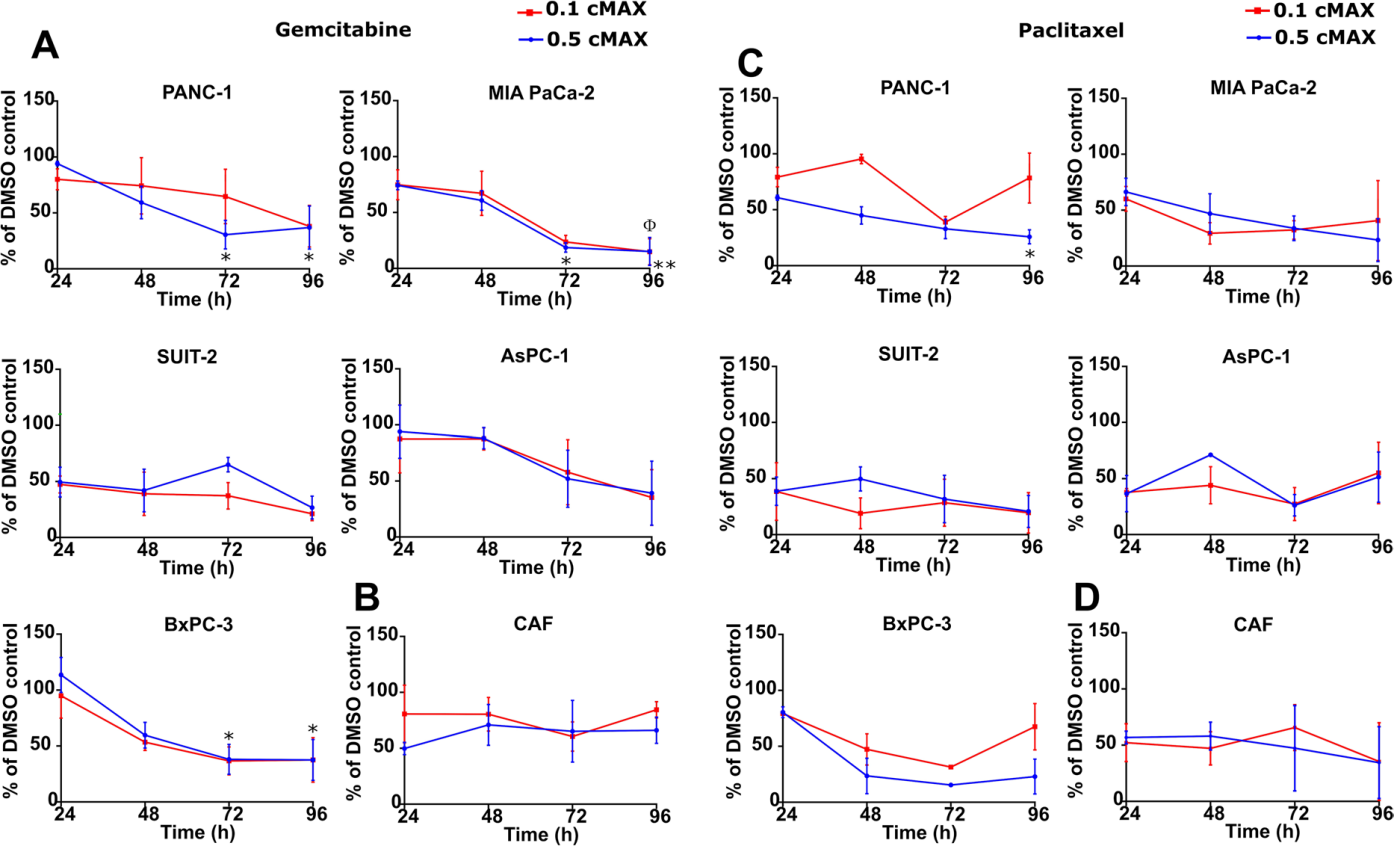
Chemotherapeutic pulsing with gemcitabine and paclitaxel to mimic clinical dosing in vitro. **(A and C)** The graphs show the cell viability of pancreatic cancer cell lines after a pulse with gemcitabine **(A)** or paclitaxel **(C)** representing 0.5 cMAX or 0.1 cMAX, data was analysed using DMSO as a control set to 100%. **(B and D)** The graphs show the cell viability of CAFs after a pulse with gemcitabine **(B)** or paclitaxel **(D)** representing 0.5 cMAX or 0.1 cMAX. At 24, 48, 72 and 96 h cell viability was determined using CellTiter-Glo. The data are shown as ± SEM of at least 2 independent experiments performed in triplicate and normalised to a DMSO control set to 100%. *P* value determined by one-way ANOVA with post hoc Dunnett’s test. The * symbol refers to the 0.5 cMAX condition with *P ≤ 0.05 and **0.01. The Φ symbol refers to the 0.1 cMAX condition with Φ *P* ≤ 0.05

Following gemcitabine treatment (Fig. 2A), all of the PDAC cell lines examined showed decreased viability when treated with either 0.1 cMAX (7.44 μM; 250 min pulse) or 0.5 cMAX (37.21 μM; 50 min pulse). MIA PaCa-2 showed the greatest sensitivity when pulsed with gemcitabine in this way, with a statistically significant reduced viability after 72 and 96 h compared to the 24 h timepoint (*P* ≤ 0.05). Panc-1 and BxPC-3 also showed a statistically significant reduced viability, whilst Suit-2 and AsPC-1 showed a similar trend of just under 40% following both 0.1 and 0.5 cMAX at 96 h. By contrast, three independent CAFs analysed (R3088, R3072, R3134) retained resistance to gemcitabine with average cell viability in excess of 60% of the DMSO control at 96 h post dosing (Fig. 2B).

PDAC cell lines showed sensitivity to paclitaxel in pulsing experiments (Fig. 2C), when treated with 0.1 cMAX (0.45 μM for 2100 min) and 0.5 cMAX (2.25 μM for 420 min) with Panc-1 reaching significance, although with the exception of Suit-2 cells, a recovery in cell viability was observed for all PDAC cells at 96 h following treatment with 0.1 cMAX. Compared to their response to gemcitabine, CAFs (R3088, R3072) were more sensitive to pulsing with paclitaxel with cell viability of 35% after 96 h pulsing with both 0.1 cMAX and 0.5 cMAX (Fig. 2D).

In summary, under the conditions of in vitro drug pulsing, all of the PDAC cell lines showed sensitivity to gemcitabine and paclitaxel. In particular, PDAC cells treated with gemcitabine showed limited signs of recovery within the 96 h experimental timeframe. Given that the partial response rate to gemcitabine of patients with advanced PDAC is 5 to 12% [40, 54], we concluded that the use of the in vitro drug pulsing protocol on its own is unlikely to minimise the discrepancy between observed effective toxicity in vitro and the poor clinical efficacy in patients. This prompted us to investigate alternative in vitro models, including the incorporation of CAFs into drug screening assays.

### CAFs reduce the anti-proliferative effect of gemcitabine in 2D co-culture models

**Fig. 3:**
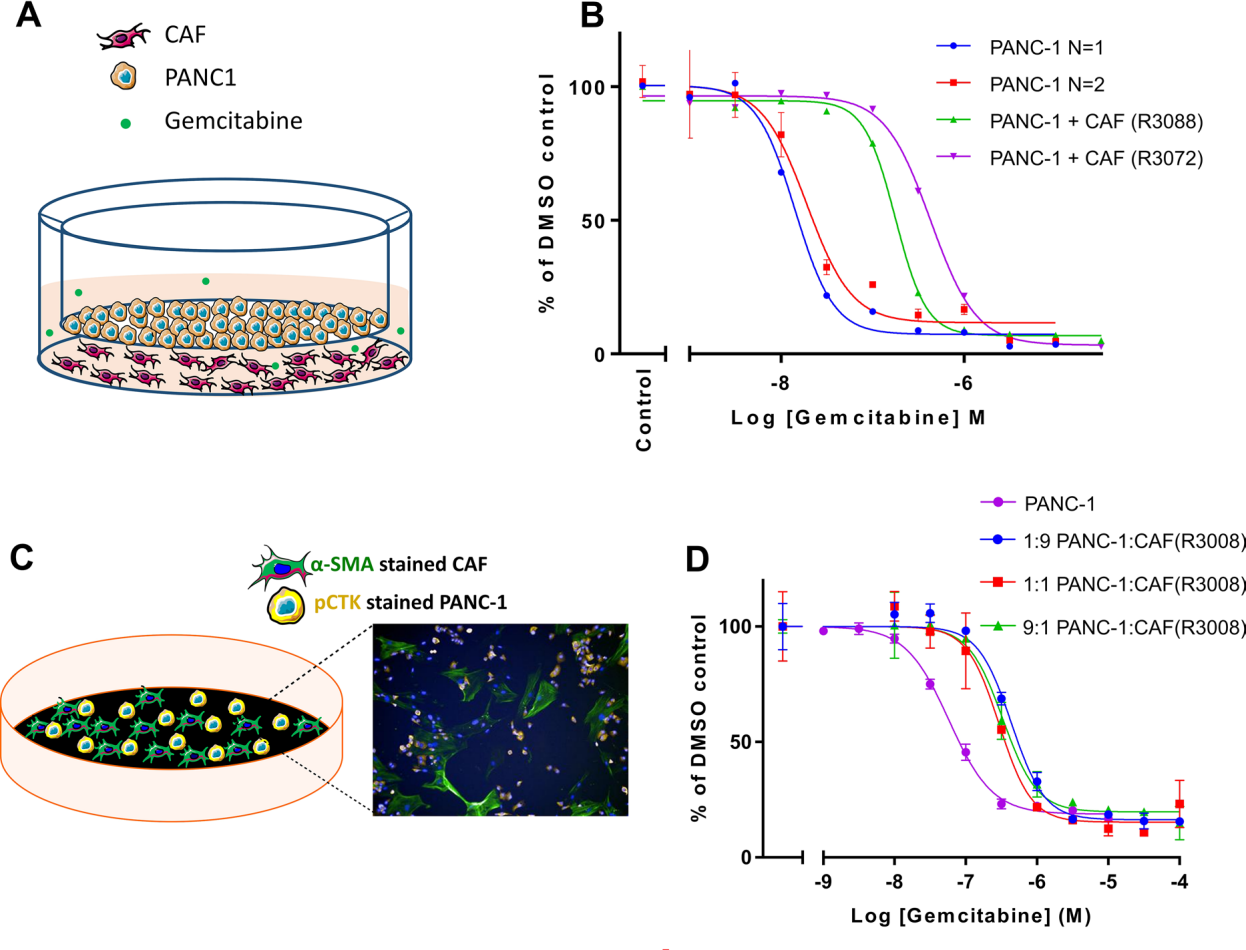
The addition of CAFs to 2D screening models reduces the anti-proliferative effect of gemcitabine on PANC-1 cells. **(A)** Schematic representation of a transwell co-culture model in which the two cell populations are separated by a physical barrier. In this model, CAFs were placed in the bottom chamber and PANC-1 cells were on the transwell insert (1:1 ratio). **(B)** Dose–response curve showing the efficacy of gemcitabine in killing PANC-1 cells in a transwell co-culture model of CAFs and PANC-1 cells. Cells were cultured for 72 h in the presence of gemcitabine. The cell viability of PANC-1 cells was measured using CellTiter-Glo. The data are shown as mean ± SD of one assay for two CAFs (R3088 and R3072) and normalised to a DMSO control set to 100%. **(C)** Schematic representation of the direct 2D co-culture model with an image depicting anti-αSMA-488 labelled CAFs (Green), anti-CTK-594-labelled PANC-1 cells (Yellow) and nuclei (Blue). The average nuclei count was measured using an Operetta (PerkinElmer), counting 4 randomly assigned areas of interest/well. **(D)** Dose–response curve showing the direct 2D cell viability assay using three different ratios of CAF to PANC-1 cells compared to PANC-1 cells alone. Cells were exposed to gemcitabine and DMSO as control. At 72 h, cell viability was determined by counting nuclei which were associated with positive pCTK staining (considered PANC1 cells) and which were not associated with areas of positive αSMA staining (considered CAFs). The data are shown as mean ± SD of one assay performed in triplicate

In order to examine whether co-culturing cancer cells with CAFs could influence the sensitivity of the cancer cells to chemotherapeutic agents, we first used a transwell co-culture model, which avoids direct cell-to-cell contact between the two distinct cell types (Fig. 3A). Two independent CAF isolates, R3088 and R3072, were evaluated for their effect on the sensitivity of PANC-1 cells to gemcitabine. The presence of R3088 CAFs in the lower chamber of the transwell plate led to a 12.5-fold increase in the IC_50_ (PANC-1 (*N* = 1) IC50 from 14.2 to 177.5 nM) (Fig. 3B). Similarly, culturing PANC-1 with R3072 CAFs led to a 22.5-fold increase in the IC_50_ (PANC-1 (*N* = 2) IC50 from 19.1 to 430.8 nM) (Fig. 3B). This suggests that the presence of CAFs reduces the ability of gemcitabine to kill cancer cells and that this effect can occur in the absence of physical contact between CAFs and cancer cells.

We next investigated whether the loss of potency of gemcitabine for PDAC cells would endure if CAFs and cancer cells were grown in a direct co-culture model in the absence of a physical barrier (Fig. 3C). Mixed cultures of PANC-1 cells and CAFs (R3088) (Fig. 3C), in varying ratios, were treated with gemcitabine for 72 h. PANC-1 cells and CAFs were then differentiated by fixing and staining with a pan-cytokeratin antibody (pCTK) and an alpha-smooth muscle actin antibody (αSMA). The viability of each cell population was determined by counting nuclei associated with distinct areas of positive staining. When compared with a mono-culture of PANC-1 cells, the addition of CAFs reduced the ability of gemcitabine to kill PANC-1 cells (Fig. 3D). This occurred regardless of the ratio of PANC-1 cells to CAFs. The exposure of PANC-1 cells alone to gemcitabine resulted in an IC50 of 58 nM, whereas the addition of CAFs caused a decrease in the sensitivity of PANC-1 to an IC50 of 331 nM (PANC-1 to CAF ratio of 9:1), 294 nM (PANC-1 to CAF ratio of 1:1) or 437 nM (PANC-1 to CAF ratio of 1:9) (Fig. 3D).

### CAFs reduce the anti-proliferative effect of gemcitabine in 2D co-culture models

**Fig. 4:**
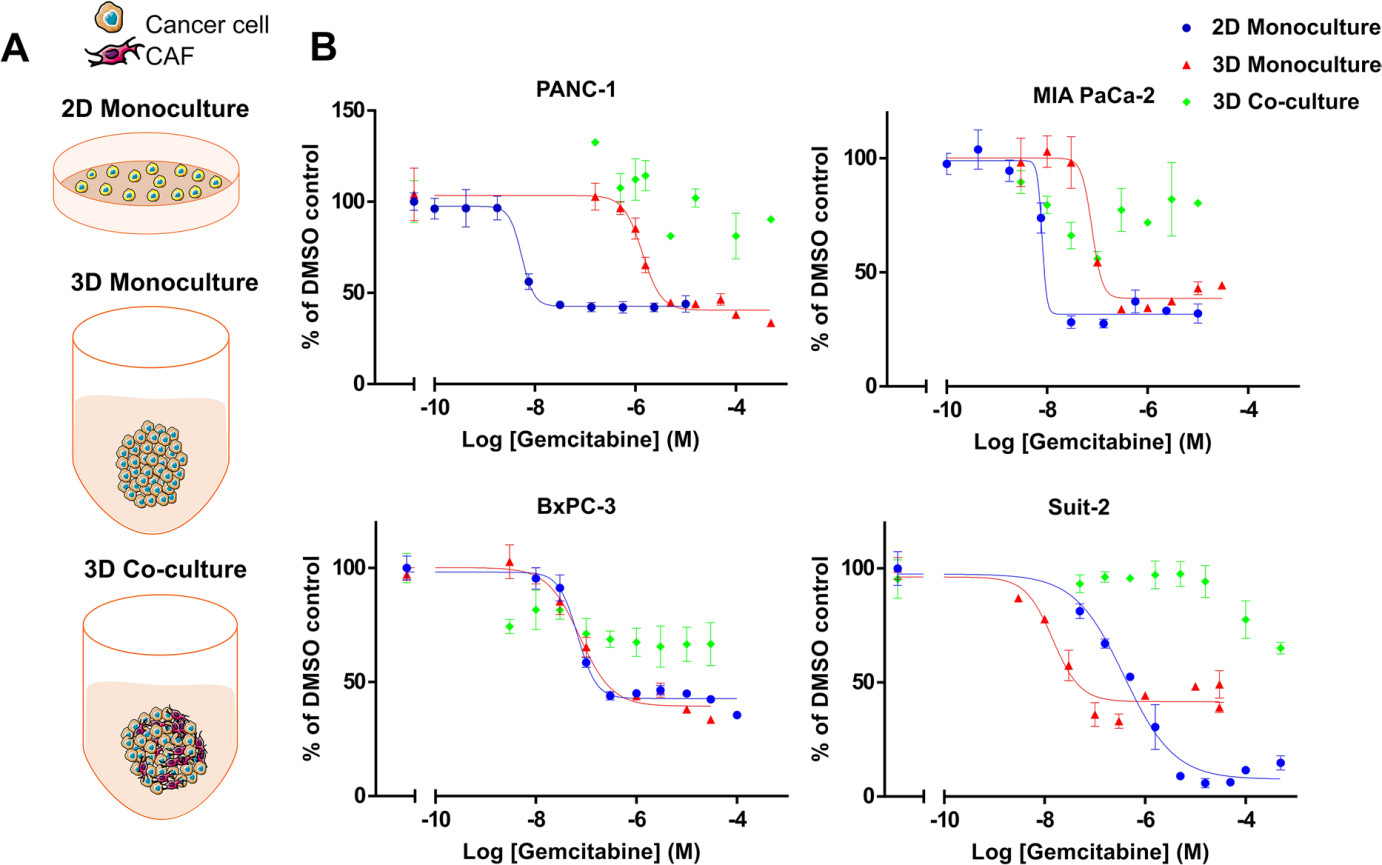
The addition of CAFs to a 3D co-culture model of pancreatic cancer cell lines confers resistance to gemcitabine. **(A)** Schematic representation of the assay formats utilised: a 2D standard mono-culture cell viability assay, a 3D mono-culture assay of pancreatic cancer cell lines and a 3D co-culture assay of pancreatic cancer cell lines combined with CAFs (R3008). **(B)** Dose–response curves of the different assay formats described above which were treated with various concentrations of gemcitabine. At 72 h cell viability was determined using CellTiter-Glo. The data are shown as ± SD of one assay performed in triplicate and normalised to a DMSO control set to 100%

Next, we sought to evaluate the efficacy of gemcitabine in a 3D co-culture model, which allows for a higher degree of structural complexity in the physical interaction between tumour cells and CAFs, and thus more closely mimics the PDAC tumour microenvironment. 3D models were established by plating cells, either as mono- or co-cultures in Ultra-Low Attachment plates where they formed spheroids (Fig. 4A). Responses to gemcitabine under these conditions were compared to a 2D mono-culture model (Fig. 4A). To determine optimal assay conditions, PANC-1 cells were cultured in 3D using 1000, 5000, 10,000, 15,000 and 20,000 cells per well and exposed to a range of concentrations of gemcitabine for 48 h, 72 h, and 96 h (Supplementary Fig. 2A). The cell density which gave the optimal assay window using PANC-1 was 1000 cells/well (Supplementary Fig. 2A). However, CAFs (R3088) were found to be minimally sensitive to gemcitabine using the 3D mono-culture model (Supplementary Fig. 2B). Following the addition of up to 30 µM of this chemotherapeutic agent, CAF cell viability did not fall below 69.5% of the DMSO control at the lowest cell density (1000 cells/well) (Supplementary Fig. 2B). The sensitivity of PANC-1 and MIA PaCa-2 cells to gemcitabine decreased when they were cultured in a 3D mono-culture model compared to 2D mono-culture (Fig. 4B). No difference in the sensitivity of BxPC-3 cells to gemcitabine was observed between these two models (Fig. 4B). However curiously, when 2D and 3D mono-cultures of Suit-2 cells were challenged with gemcitabine, the 3D cultures proved to be more sensitive than the 2D cultures.

For 3D co-culture experiments, a ratio of 1:1 PDAC cells to CAFs (R3088) was used as this had been effective in the in-direct and direct 2D co-culture models tested (Fig. 3B, D). The addition of CAFs to 3D models of PANC1, Suit-2 and BxPC3 was accompanied by greater cell viability, despite the use of concentrations of gemcitabine up to 50 µM. Using 50 µM gemcitabine, the total cell viability of 3D co-cultured CAFs and PANC-1 cells was 90.3% (compared to vehicle-treated controls) versus 33.5% for the PANC1 3D mono-culture (compared to vehicle controls). Similarly, Suit-2 cells in the 3D co-culture model showed a cell viability of 65% when treated with 50 µM of gemcitabine (compared to vehicle controls) versus 39% at 30 µM gemcitabine in the 3D mono-culture, and BxPC3 cells in the 3D co-culture showed a cell viability of 66.6% compared to 33.5% in the 3D mono-culture at a concentration of 30 µM gemcitabine. Although using MIA PaCa-2 cells in the 3D co-culture model resulted in variable cell viability, a loss of potency of gemcitabine was nonetheless observed (Fig. 4B).

The potency of paclitaxel was also compared in 2D and 3D mono-culture in addition to 3D co-culture assays (Supplementary Fig. 3). For MIA PaCa-2 cells, there was no measurable difference in the potency of paclitaxel between cells cultured in any of the models tested. Similarly, PANC-1 cells showed little change in the IC_50_ values when comparing the three assays. In the case of Suit-2 cells, both the 3D and 2D mono-culture assays gave similar IC_50_ values (7.8 and 3.1 nM respectively). In the Suit-2 3D co-culture assay, it was not possible to determine an IC_50_ value due to the shape of the curve; however, the cell viability at 100 µM paclitaxel was approximately 45% (compared to vehicle control treated cells) versus 32% in the Suit-2 3D mono-culture assay at 30 µM paclitaxel. It was not possible to determine an IC_50_ value for BxPC3 cells cultured with CAFs in the 3D co-culture model as inhibition of cell viability reached only approximately 70%. Taken together, these data provide further evidence that CAFs provide a margin of chemoresistance that is not observed in PDAC cell line mono-cultures and that their incorporation into models in drug discovery deserves further consideration.

## Discussion

The marked discrepancy between in vitro cytotoxicity studies showing effectiveness of gemcitabine or paclitaxel against pancreatic cancer cells [1, 8] and in vivo or clinical trial studies which show only small survival gains from these chemotherapies [3, 11] raises important questions about the reliability of cell based pre-clinical testing. Profiling the anti-proliferative potencies of new therapies using IC_50_ values obtained from 2D monolayer assays [9, 37] is well established, inexpensive and reproducible [29, 32]. However, limitations include the lack of consideration of dosing schedule and the potential contribution of non-cancer cells of the tumour microenvironment to treatment response. In relation to dosing schedule, we observed that pancreatic cancer cells demonstrated sensitivity to both gemcitabine and paclitaxel in an in vitro drug pulsing assay, suggesting that this protocol was unlikely to minimise the discrepancy between observed effective toxicity in vitro and poor clinical efficacy. We therefore turned our attention to the incorporation of CAFs into drug screening assays.

We firstly ascertained that CAFs, isolated from three surgically resected PDAC tissue samples, were functionally active as demonstrated by their secretion of collagen 1A1, in agreement with their myofibroblast role in regulating collagen fibres in the tumour microenvironment [5, 22]. Unlike PDAC cell lines, our isolated CAFs displayed intrinsic resistance to gemcitabine and paclitaxel, consistent with previous studies in PDAC [49] and in breast and lung cancers [53].

Diminished gemcitabine potency for PDAC cells was observed under all conditions where PDAC and CAFs were co-cultured. Our transwell co-culture model highlighted that physical contact between CAFs and PDAC cells was not a prerequisite for the reduced gemcitabine cytotoxicity. Hessmann et al. similarly observed that conditioned media of murine CAFs pre-incubated with gemcitabine led to a 40–80% increment in the cell viability of KPC cell lines in comparison to fresh addition of gemcitabine to CAF-conditioned media, attributing this effect to gemcitabine scavenging properties of CAFs in vitro [23]. Such a fibroblast-dependent protective effect is consistent with the observation that addition of NIH-3T3 fibroblasts to a transwell co-culture with BxPC3 cells reduced the effect of doxorubicin on BxPC3 cells, which could be rescued by the addition of smoothened inhibitor vismodegib indicating that paracrine Hh signalling was active in the co-culture [61]. Taken together, this indicates that CAFs provide a chemotherapeutic-resistance mechanism to PDAC cells which is not dependent upon physical contact between CAFs and PDAC cells, suggesting that scavenging of gemcitabine and/or a paracrine signalling pathway are significant contributing factors. This is supported by the observation that paracrine signalling is activated by the CAF secretome which has a role in chemoresistance such as mTOR/4E-BP1 [14] and SDF-1α [60].

Using direct co-culture models, we observed a similar loss of gemcitabine potency when the cells were allowed to form physical connections. Such reduction in potency has also been observed in SW480 colon cancer cells in co-culture with WI-38 fibroblasts in the presence of the WNT/β-catenin signalling inhibitor XAV939 [31]. Cell–cell contact between CAFs and squamous cell carcinoma cells has been found to play a role in the invasion and migration of the tumour cells, through ECM remodelling [18]. This indicates that both paracrine and physical communication between CAFs and tumour cells are important in the tumour microenvironment.

Utilising a 3D co-culture model, we found that culturing tumour cells in the presence of CAFs reduced the potency of gemcitabine more than in a 2D or 3D mono-culture model. These results are in agreement with Lee et al. who used a microfluidic channel plate to embed co-culture spheroids of pancreatic tumour cell lines and CAFs into a collagen matrix and found that co-cultures resulted in increased drug resistance [34]. In head and neck cancers (HNC), 3D spheroids containing co-cultures of HNC cell lines with CAFs showed greater invasiveness than 3D mono-cultures of HNC cell lines into the fibrin matrix of a 3D cell sheet containing oral keratinocytes, fibroblasts and plasma fibrin. In addition, enhanced resistance to cisplatin and sorafenib was more easily observed in 3D spheroids and CAFs than in 2D models [33].

The loss of potency of paclitaxel was observed in 50% only of the cell lines tested in our 3D co-culture model implying that different mechanisms of CAF-mediated resistance are in place depending on the cancer cell line employed. In support of this notion, Marusyk et al. (2016) identified variable levels of resistance to paclitaxel and doxorubicin amongst different breast cancer cell lines co-cultured with CAFs. However, the protection of carcinoma cells by fibroblasts against a different chemotherapeutic drug, lapatinib, was observed more consistently in all the cell lines studied [39]. With respect to mechanism, PDAC cell lines [15] and ovarian tumour cells [58] treated with conditioned medium from CAFs were shown to have increased resistance to paclitaxel, attributed to the presence of IL-6 secreted by the CAFs which has been found to promote survival in tumour cells. Triple-negative breast cancer cells grown in a 3D co-culture with CAFs showed increased resistance to treatment with paclitaxel. This was found to be due to CXCL12-CXCR4 paracrine signalling between the CAFs and tumour cells resulting in activation of the MAPK/PI3K pathways [21].

Culture conditions impart significant functional characteristics on cells. 3D models have been suggested as the future of pharmacological drug screening assays [28] as they recapitulate some aspects of a tumour such as allowing cells to retain a 3D structure [12], the presence of a nutrient gradient, cell junctions [32] and polarity [35]. In a 3D model, the cells have less access to the drugs and undergo changes in cell cycle as well as increased hypoxic conditions. All of these factors have been reported to reduce sensitivity to chemotherapies [29]. In addition, it has been found that 3D cell models which have a density comparable to that of tissue will more accurately predict the response to drugs [26]. The data discussed herein adds further evidence to the importance of culture conditions when testing chemotherapeutic agents.

Collectively, this study provides evidence that the PDAC tumour microenvironment is dynamic in its response to chemotherapeutic agents, and CAFs play a much more elaborate role in chemotherapeutic resistance than just providing a physical barrier. Models such as the 3D and CAF/PDAC co-culture methods used in this study offer an avenue to bridge that gap between in vitro and in vivo testing, especially in the case of PDAC in which the interactions between CAFs and tumour cells significantly impact responses to therapy. Future studies should investigate other drugs relevant to pancreatic cancer, such as 5-FU and SN38.

## Supplementary Information

Below is the link to the electronic supplementary material.

Supplementary file 1 Pancreatic cancer cell lines were sensitive to gemcitabine and paclitaxel in standard 2D culture conditions after 96h treatment (A and B) Dose-response curve of a 2D cell viability assay. Pancreatic cancer cell lines are sensitive to gemcitabine (A) and paclitaxel (B). Cells were exposed to increasing concentrations of gemcitabine or paclitaxel, and cell viability measured at 96h using CellTiter-Glo. Data were fitted to a sigmoidal dose response curve and IC50 was determined using GraphPad prism. The data are shown as ±SD of one assay performed in triplicate and normalised to a DMSO control set to 100%. (C and D) Graphs depicting a comparison of the mean pIC50 in molar (M) ±S.E.M for multiple assays of gemcitabine (C) and paclitaxel (D) in pancreatic cancer cell lines (JPG 4377 KB)

Supplementary file 2 Establishment of optimal conditions for measuring cell viability in a 3D mono-culture model of Panc-1 or CAFs (A) Dose-response curves showing the response of PANC-1 cells cultured in 3D to gemcitabine using different cell numbers over a range of time-points to determine an optimal assay window. (B) Dose-response curves showing the response of CAFs (R3008) cultured at a variety of cell densities in 3D to gemcitabine treatment for 72h. Cell viability was determined using CellTiter-Glo at the specified time point and normalised to a DMSO control set to 100% (JPG 2453 KB)

Supplementary file 3 The addition of CAFs to a 3D co-culture model with pancreatic cancer cell lines impacts the efficacy of paclitaxel. Graphs show the comparison of a 2D standard mono-culture cell viability assay, a 3D mono-culture assay of pancreatic cancer cell lines and a 3D co-cultures assay of pancreatic cancer cell lines combined with CAFs, which were treated with various concentrations of paclitaxel. At 72h cell viability was determined using CellTiter-Glo. The data are shown as ±SD of one assay performed in triplicate and normalised to a DMSO control set to 100% (JPG 2699 KB)

## References

[1] Alber, M. S., Lee, J. J., Huang, J., England, C. G., McNally, L. R., & Frieboes, H. B. (2013). Predictive modeling of in vivo response to gemcitabine in pancreatic cancer. PLoS Computational Biology, 9(9). 10.1371/journal.pcbi.1003231 10.1371/journal.pcbi.1003231 PMC3777914 24068909

[2] Apte MV, Haber PS, Applegate TL, Norton ID, McCaughan GW, Korsten MA, Pirola RC, Wilson JS (1998). Periacinar stellate shaped cells in rat pancreas: identification, isolation, and culture. Gut.

[3] Awasthi N, Zhang C, Schwarz AM, Hinz S, Wang C, Williams NS, Schwarz MA, Schwarz RE (2013). Comparative benefits of Nab-paclitaxel over gemcitabine or polysorbate-based docetaxel in experimental pancreatic cancer. Carcinogenesis.

[4] Bachem MG, Schneider E, Gross H, Weidenbach H, Schmid RM, Menke A, Siech M, Beger H, Grunert A, Adler G (1998). Identification, culture, and characterization of pancreatic stellate cells in rats and humans. Gastroenterology.

[5] Bachem MG, Schunemann M, Ramadani M, Siech M, Beger H, Buck A, Zhou S, Schmid-Kotsas A, Adler G (2005). Pancreatic carcinoma cells induce fibrosis by stimulating proliferation and matrix synthesis of stellate cells. Gastroenterology.

[6] Barati Bagherabad M, Afzaljavan F, ShahidSales S, Hassanian SM, Avan A (2019). Targeted therapies in pancreatic cancer: promises and failures. J Cell Biochem.

[7] Barrera LN, Evans A, Lane B, Brumskill S, Oldfield FE, Campbell F, Andrews T, Lu Z, Perez-Mancera PA, Liloglou T, Ashworth M, Jalali M, Dawson R, Nunes Q, Phillips PA, Timms JF, Halloran C, Greenhalf W, Neoptolemos JP, Costello E (2020). Fibroblasts from distinct pancreatic pathologies exhibit disease-specific properties. Cancer Res.

[8] Braun, L. M., Lagies, S., Guenzle, J., Fichtner-Feigl, S., Wittel, U. A., & Kammerer, B. (2020). Metabolic adaptation during nab-paclitaxel resistance in pancreatic cancer cell lines. Cells, 9(5). 10.3390/cells9051251 10.3390/cells9051251 PMC7290296 32438599

[9] Breslin S, O'Driscoll L (2013). Three-dimensional cell culture: the missing link in drug discovery. Drug Discov Today.

[10] Carmichael J, Fink U, Russell RC, Spittle MF, Harris AL, Spiessi G, Blatter J (1996). Phase II study of gemcitabine in patients with advanced pancreatic cancer. Br J Cancer.

[11] Corrie PG, Qian W, Basu B, Valle JW, Falk S, lwuji C, Wasan H, Palmer D, Scott-Brown M, Wadsley J, Arif S, Bridgewater J, Propper D, Gillmore R, Gopinathan A, Skells R, Bundi P, Brais R, Dalchau K, Bax L, Chhabra A, Machin A, Dayim A, McAdam K, Cummins S, Wall L, Ellis R, Anthoney A, Evans J, Ma YT, Isherwood C, Neesse A, Tuveson D, Jodrell DI (2020). Scheduling nab-paclitaxel combined with gemcitabine as first-line treatment for metastatic pancreatic adenocarcinoma. Br J Cancer.

[12] Costa EC, Moreira AF, de Melo-Diogo D, Gaspar VM, Carvalho MP, Correia IJ (2016). 3D tumor spheroids: an overview on the tools and techniques used for their analysis. Biotechnol Adv.

[13] CRUK. (2020). Pancreatic cancer mortality (Cancer Research UK). Retrieved from https://www.cancerresearchuk.org/health-professional/cancer-statistics/statistics-by-cancer-type/pancreatic-cancer

[14] Duluc C, Moatassim-Billah S, Chalabi-Dchar M, Perraud A, Samain R, Breibach F, Gayral M, Cordelier P, Delisle MB, Bousquet-Dubouch MP, Tomasini R, Schmid H, Mathonnet M, Pyronnet S, Martineau Y, Bousquet C (2015). Pharmacological targeting of the protein synthesis mTOR/4E-BP1 pathway in cancer-associated fibroblasts abrogates pancreatic tumour chemoresistance. EMBO Mol Med.

[15] Feng R, Morine Y, Ikemoto T, Imura S, Iwahashi S, Saito Y, Shimada M (2018). Nab-paclitaxel interrupts cancer-stromal interaction through C-X-C motif chemokine 10-mediated interleukin-6 downregulation in vitro. Cancer Sci.

[16] Fogli S, Danesi R, Gennari A, Donati S, Conte PF, Del Tacca M (2002). Gemcitabine, epirubicin and paclitaxel: pharmacokinetic and pharmacodynamic interactions in advanced breast cancer. Ann Oncol.

[17] Froeling FE, Marshall JF, Kocher HM (2010). Pancreatic cancer organotypic cultures. J Biotechnol.

[18] Gaggioli C, Hooper S, Hidalgo-Carcedo C, Grosse R, Marshall JF, Harrington K, Sahai E (2007). Fibroblast-led collective invasion of carcinoma cells with differing roles for RhoGTPases in leading and following cells. Nat Cell Biol.

[19] Gorchs L, Fernandez Moro C, Bankhead P, Kern KP, Sadeak I, Meng Q, Rangelova E, Kaipe H (2019). Human pancreatic carcinoma-associated fibroblasts promote expression of co-inhibitory markers on CD4(+) and CD8(+) T-cells. Front Immunol.

[20] Haber PS, Keogh GW, Apte MV, Moran CS, Stewart NL, Crawford DH, Pirola RC, McCaughan GW, Ramm GA, Wilson JS (1999). Activation of pancreatic stellate cells in human and experimental pancreatic fibrosis. Am J Pathol.

[21] Ham SL, Thakuri PS, Plaster M, Li J, Luker KE, Luker GD, Tavana H (2018). Three-dimensional tumor model mimics stromal - breast cancer cells signaling. Oncotarget.

[22] Hanley CJ, Noble F, Ward M, Bullock M, Drifka C, Mellone M, Manousopoulou A, Johnston HE, Hayden A, Thirdborough S, Liu Y, Smith DM, Mellows T, Kao WJ, Garbis SD, Mirnezami A, Underwood TJ, Eliceiri KW, Thomas GJ (2016). A subset of myofibroblastic cancer-associated fibroblasts regulate collagen fiber elongation, which is prognostic in multiple cancers. Oncotarget.

[23] Hessmann E, Patzak MS, Klein L, Chen N, Kari V, Ramu I, Bapiro TE, Frese KK, Gopinathan A, Richards FM, Jodrell DI, Verbeke C, Li X, Heuchel R, Lohr JM, Johnsen SA, Gress TM, Ellenrieder V, Neesse A (2018). Fibroblast drug scavenging increases intratumoural gemcitabine accumulation in murine pancreas cancer. Gut.

[24] Horvath P, Aulner N, Bickle M, Davies AM, Nery ED, Ebner D, Montoya MC, Ostling P, Pietiainen V, Price LS, Shorte SL, Turcatti G, von Schantz C, Carragher NO (2016). Screening out irrelevant cell-based models of disease. Nat Rev Drug Discov.

[25] Hosein AN, Brekken RA, Maitra A (2020). Pancreatic cancer stroma: an update on therapeutic targeting strategies. Nat Rev Gastroenterol Hepatol.

[26] Hsieh CH, Chen YD, Huang SF, Wang HM, Wu MH (2015). The effect of primary cancer cell culture models on the results of drug chemosensitivity assays: the application of perfusion microbioreactor system as cell culture vessel. Biomed Res Int.

[27] Hughes JP, Rees S, Kalindjian SB, Philpott KL (2011). Principles of early drug discovery. Br J Pharmacol.

[28] Jensen C, Teng Y (2020). Is it time to start transitioning from 2D to 3D cell culture?. Front Mol Biosci.

[29] Kapalczynska M, Kolenda T, Przybyla W, Zajaczkowska M, Teresiak A, Filas V, Ibbs M, Blizniak R, Luczewski L, Lamperska K (2018). 2D and 3D cell cultures—a comparison of different types of cancer cell cultures. Arch Med Sci.

[30] Kleeff J, Korc M, Apte M, La Vecchia C, Johnson CD, Biankin AV, Neale RE, Tempero M, Tuveson DA, Hruban RH, Neoptolemos JP (2016). Pancreatic cancer Nat Rev Dis Primers.

[31] Koh B, Jeon H, Kim D, Kang D, Kim KR (2019). Effect of fibroblast co-culture on the proliferation, viability and drug response of colon cancer cells. Oncol Lett.

[32] Langhans SA (2018). Three-dimensional in vitro cell culture models in drug discovery and drug repositioning. Front Pharmacol.

[33] Lee J, Shin D, Roh JL (2018). Development of an in vitro cell-sheet cancer model for chemotherapeutic screening. Theranostics.

[34] Lee JH, Kim SK, Khawar IA, Jeong SY, Chung S, Kuh HJ (2018). Microfluidic co-culture of pancreatic tumor spheroids with stellate cells as a novel 3D model for investigation of stroma-mediated cell motility and drug resistance. J Exp Clin Cancer Res.

[35] Lelievre SA, Kwok T, Chittiboyina S (2017). Architecture in 3D cell culture: an essential feature for in vitro toxicology. Toxicol In Vitro.

[36] Lovitt CJ, Shelper TB, Avery VM (2014). Advanced cell culture techniques for cancer drug discovery. Biology (Basel).

[37] Lv D, Hu Z, Lu L, Lu H, Xu X (2017). Three-dimensional cell culture: a powerful tool in tumor research and drug discovery. Oncol Lett.

[38] Ma WW, Hidalgo M (2013). The winning formulation: the development of paclitaxel in pancreatic cancer. Clin Cancer Res.

[39] Marusyk A, Tabassum DP, Janiszewska M, Place AE, Trinh A, Rozhok AI, Pyne S, Guerriero JL, Shu S, Ekram M, Ishkin A, Cahill DP, Nikolsky Y, Chan TA, Rimawi MF, Hilsenbeck S, Schiff R, Osborne KC, Letai A, Polyak K (2016). Spatial proximity to fibroblasts impacts molecular features and therapeutic sensitivity of breast cancer cells influencing clinical outcomes. Can Res.

[40] Min YJ, Joo KR, Park NH, Yun TK, Nah YW, Nam CW, Park JH (2002). Gemcitabine therapy in patients with advanced pancreatic cancer. Korean J Intern Med.

[41] Moore M (1996). Activity of gemcitabine in patients with advanced pancreatic carcinoma. A review. Cancer.

[42] Neesse A, Algul H, Tuveson DA, Gress TM (2015). Stromal biology and therapy in pancreatic cancer: a changing paradigm. Gut.

[43] Norberg, K. J., Liu, X., Fernández Moro, C., Strell, C., Nania, S., Blümel, M., Balboni, A., Bozóky, B., Heuchel, R. L., & Löhr, J. M. (2020). A novel pancreatic tumour and stellate cell 3D co-culture spheroid model. BMC Cancer, 20(1). 10.1186/s12885-020-06867-5 10.1186/s12885-020-06867-5 PMC7251727 32460715

[44] Olive KP (2015). Stroma, Stroma everywhere (far more than you think). Clin Cancer Res.

[45] Olive, K. P., Jacobetz, M. A., Davidson, C. J., Gopinathan, A., McIntyre, D., Honess, D., Madhu, B., Goldgraben, M. A., Caldwell, M. E., Allard, D., Frese, K. K., Denicola, G., Feig, C., Combs, C., Winter, S. P., Ireland-Zecchini, H., Reichelt, S., Howat, W. J., Chang, A., Dhara, M., Wang, L., Ruckert, F., Grutzmann, R., Pilarsky, C., Izeradjene, K., Hingorani, S. R., Huang, P., Davies, S. E., Plunkett, W., Egorin, M., Hruban, R. H., Whitebread, N., McGovern, K., Adams, J., Iacobuzio-Donahue, C., Griffiths, J., & Tuveson, D. A. (2009). Inhibition of Hedgehog signaling enhances delivery of chemotherapy in a mouse model of pancreatic cancer. Science, 324(5933), 1457-146110.1126/science.1171362 10.1126/science.1171362 PMC2998180 19460966

[46] Provenzano PP, Cuevas C, Chang AE, Goel VK, Von Hoff DD, Hingorani SR (2012). Enzymatic targeting of the stroma ablates physical barriers to treatment of pancreatic ductal adenocarcinoma. Cancer Cell.

[47] Provenzano PP, Hingorani SR (2013). Hyaluronan, fluid pressure, and stromal resistance in pancreas cancer. Br J Cancer.

[48] Rawla P, Sunkara T, Gaduputi V (2019). Epidemiology of pancreatic cancer: global trends, etiology and risk factors. World J Oncol.

[49] Richards KE, Zeleniak AE, Fishel ML, Wu J, Littlepage LE, Hill R (2017). Cancer-associated fibroblast exosomes regulate survival and proliferation of pancreatic cancer cells. Oncogene.

[50] Roth, M. T., Cardin, D. B., & Berlin, J. D. (2020). Recent advances in the treatment of pancreatic cancer. F1000Res, 9. 10.12688/f1000research.21981.1 10.12688/f1000research.21981.1 PMC7043109 32148767

[51] Sherman MH, Yu RT, Tseng TW, Sousa CM, Liu S, Truitt ML, He N, Ding N, Liddle C, Atkins AR, Leblanc M, Collisson EA, Asara JM, Kimmelman AC, Downes M, Evans RM (2017). Stromal cues regulate the pancreatic cancer epigenome and metabolome. Proc Natl Acad Sci U S A.

[52] Siegel RL, Miller KD, Jemal A (2019). Cancer statistics, 2019. CA Cancer J Clin.

[53] Sonnenberg M, van der Kuip H, Haubeis S, Fritz P, Schroth W, Friedel G, Simon W, Murdter TE, Aulitzky WE (2008). Highly variable response to cytotoxic chemotherapy in carcinoma-associated fibroblasts (CAFs) from lung and breast. BMC Cancer.

[54] Terashima T, Yamashita T, Sakai A, Ohta H, Hinoue Y, Toya D, Kawai H, Yonejima M, Urabe T, Noda Y, Mizukoshi E, Kaneko S (2018). Treatment patterns and outcomes of unresectable pancreatic cancer patients in real-life practice: a region-wide analysis. Jpn J Clin Oncol.

[55] Thota R, Maitra A, Berlin JD (2017). Preclinical rationale for the phase III trials in metastatic pancreatic cancer: is wishful thinking clouding successful drug development for pancreatic cancer?. Pancreas.

[56] Uzunparmak B, Sahin IH (2019). Pancreatic cancer microenvironment: a current dilemma. Clin Transl Med.

[57] Von Hoff DD, Ervin T, Arena FP, Chiorean EG, Infante J, Moore M, Seay T, Tjulandin SA, Ma WW, Saleh MN, Harris M, Reni M, Dowden S, Laheru D, Bahary N, Ramanathan RK, Tabernero J, Hidalgo M, Goldstein D, Van Cutsem E, Wei X, Iglesias J, Renschler MF (2013). Increased survival in pancreatic cancer with nab-paclitaxel plus gemcitabine. N Engl J Med.

[58] Wang L, Zhang F, Cui JY, Chen L, Chen YT, Liu BW (2018). CAFs enhance paclitaxel resistance by inducing EMT through the IL-6/JAK2/STAT3 pathway. Oncol Rep.

[59] Whatcott CJ, Diep CH, Jiang P, Watanabe A, LoBello J, Sima C, Hostetter G, Shepard HM, Von Hoff DD, Han H (2015). Desmoplasia in primary tumors and metastatic lesions of pancreatic cancer. Clin Cancer Res.

[60] Zhang H, Wu H, Guan J, Wang L, Ren X, Shi X, Liang Z, Liu T (2015). Paracrine SDF-1alpha signaling mediates the effects of PSCs on GEM chemoresistance through an IL-6 autocrine loop in pancreatic cancer cells. Oncotarget.

[61] Zhou Q, Zhou Y, Liu X, Shen Y (2017). GDC-0449 improves the antitumor activity of nano-doxorubicin in pancreatic cancer in a fibroblast-enriched microenvironment. Sci Rep.

