# Supplementary figures and images for: Inclusion of cancer-associated fibroblasts in drug screening assays to evaluate pancreatic cancer resistance to therapeutic drugs
Source: J Physiol Biochem. 2021 Dec 5;79(1):223–34. doi: 10.1007/s13105-021-00857-2 (PMC9905179; doi:10.1007/s13105-021-00857-2)

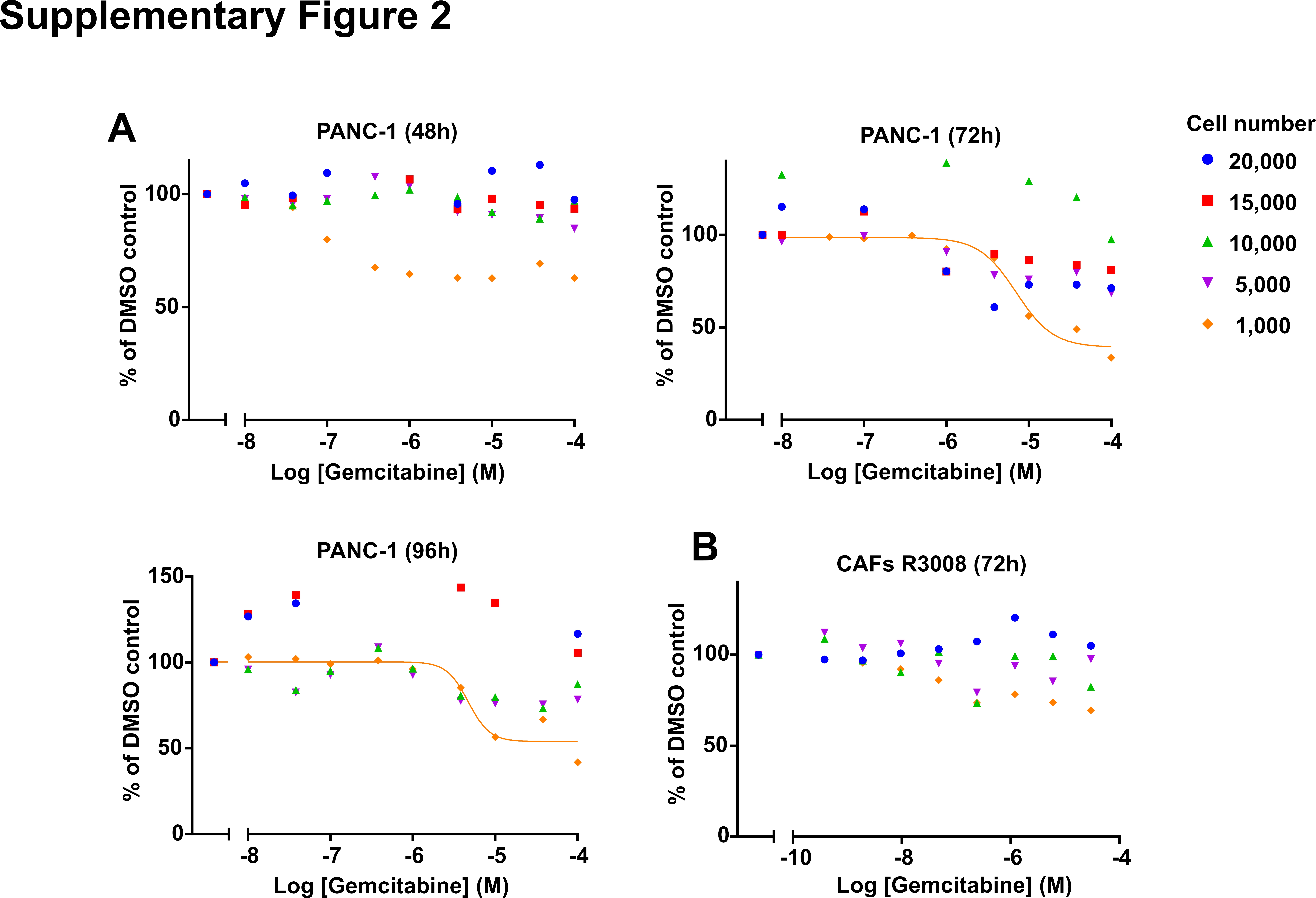

Supplement: Supplementary file 2 — Establishment of optimal conditions for measuring cell viability in a 3D mono-culture model of Panc-1 or CAFs (A) Dose-response curves showing the response of PANC-1 cells cultured in 3D to gemcitabine using different cell numbers over a range of time-points to determine an optimal assay window. (B) Dose-response curves showing the response of CAFs (R3008) cultured at a variety of cell densities in 3D to gemcitabine treatment for 72h. Cell viability was determined using CellTiter-Glo at the specified time point and normalised to a DMSO control set to 100% (JPG 2453 KB) [file 13105_2021_857_MOESM2_ESM.jpg]

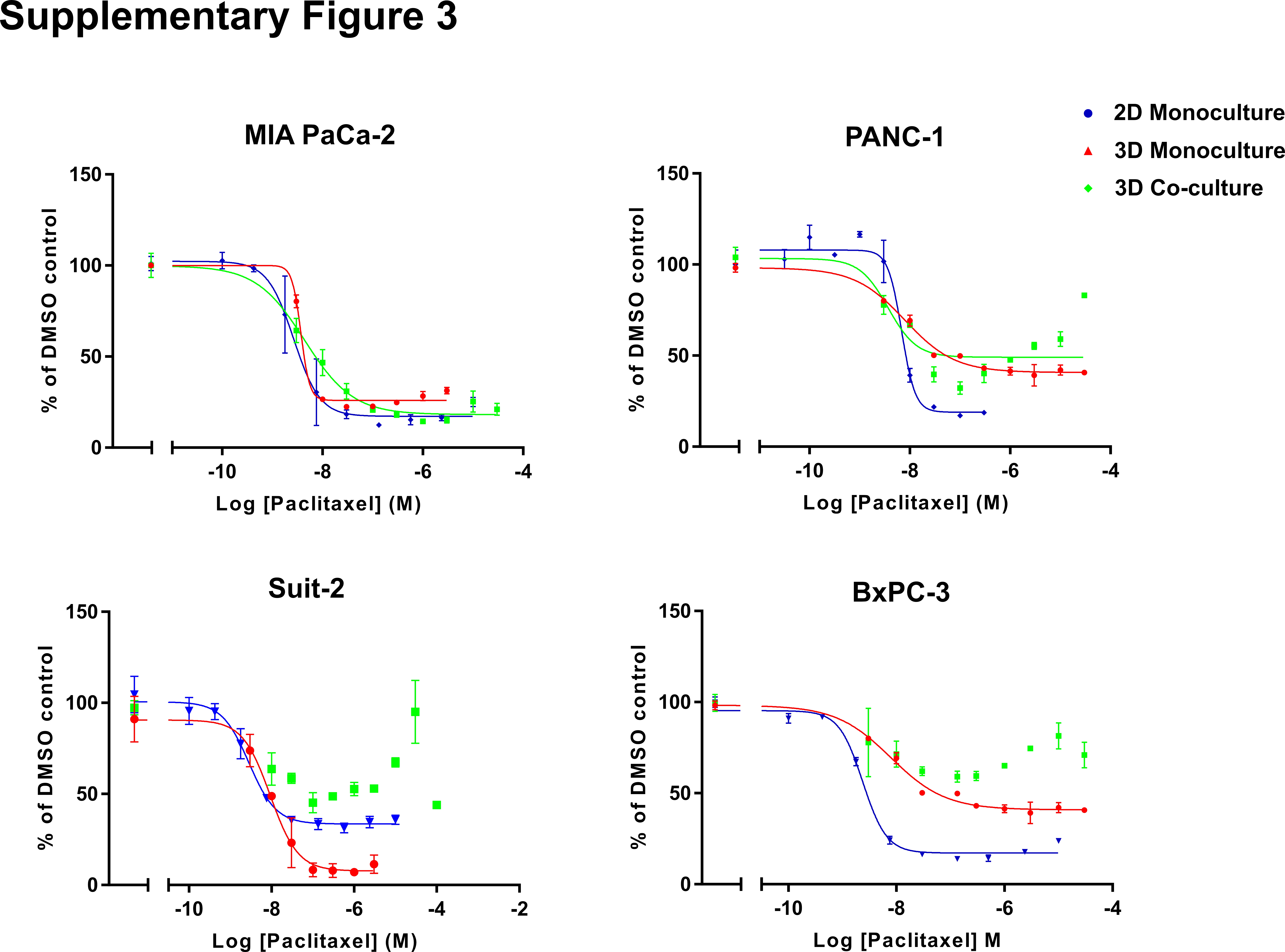

Supplement: Supplementary file 3 — The addition of CAFs to a 3D co-culture model with pancreatic cancer cell lines impacts the efficacy of paclitaxel. Graphs show the comparison of a 2D standard mono-culture cell viability assay, a 3D mono-culture assay of pancreatic cancer cell lines and a 3D co-cultures assay of pancreatic cancer cell lines combined with CAFs, which were treated with various concentrations of paclitaxel. At 72h cell viability was determined using CellTiter-Glo. The data are shown as ±SD of one assay performed in triplicate and normalised to a DMSO control set to 100% (JPG 2699 KB) [file 13105_2021_857_MOESM3_ESM.jpg]
